# Classical Biomarker and Quantitative Extended Diamondoid Analysis Fingerprints for Crude Oils from Deepwater Developments in Block 17, Lower Congo Basin, Angola

**DOI:** 10.3390/ijerph17197204

**Published:** 2020-10-01

**Authors:** Carlos Boente, Gonzalo Márquez, Patricia Marín, Emilio Romero, Cristina Rodrigues, Marco Antonio Guzmán

**Affiliations:** 1 Department of Mining, Mechanical, Energetic and Civil Engineering, University of Huelva, 21819 Huelva, Spain; gonzalo.marquez@diq.uhu.es (G.M.); romaci@uhu.es (E.R.); marcoantonioguzmanvillanueva@gmail.com (M.A.G.); 2 Department of Geochemistry, Petrology and Geological Exploration, University of Barcelona, 08028 Barcelona, Spain; p.marinbarba@gmail.com; 3 Energy, Environment and Health Research Unity, University Fernando Pessoa, 4249-004 Porto, Portugal; cfrodrig@gmail.com

**Keywords:** Block 17, mixed oils, QEDA analysis, PM biodegradation scale, Lower Congo Basin

## Abstract

The organic geochemistry of six oil samples from the offshore Block 17 (Lower Congo Basin, northwestern Angola) was studied by a combination of classical biomarker and extended diamondoid analyses to elucidate source rock facies, the extent of biodegradation, and thermal maturity. Based on molecular data, oils are interpreted as depicting a mixture of two pulses of hydrocarbon generation probably from the Bucomazi and Malembo formations. Geochemical results also gave evidence of mixing of a lacustrine siliciclastic-sourced oil charge and a second more terrestrially derived oil type in the samples analyzed. A single genetic oil family was identified through hierarchical cluster analysis; however, two groups of oils were identified on the basis of their biodegradation levels using the Peters/Moldowan scale. Lower and upper Malembo oils have a slight depletion and a notable absence of *n*-alkanes, suggesting PM levels of 1 and 2, respectively. Most molecular maturity parameters of the oil samples suggest a maturity level equivalent to the onset of the peak of the oil generative window.

## 1. Introduction

**Figure 1:**
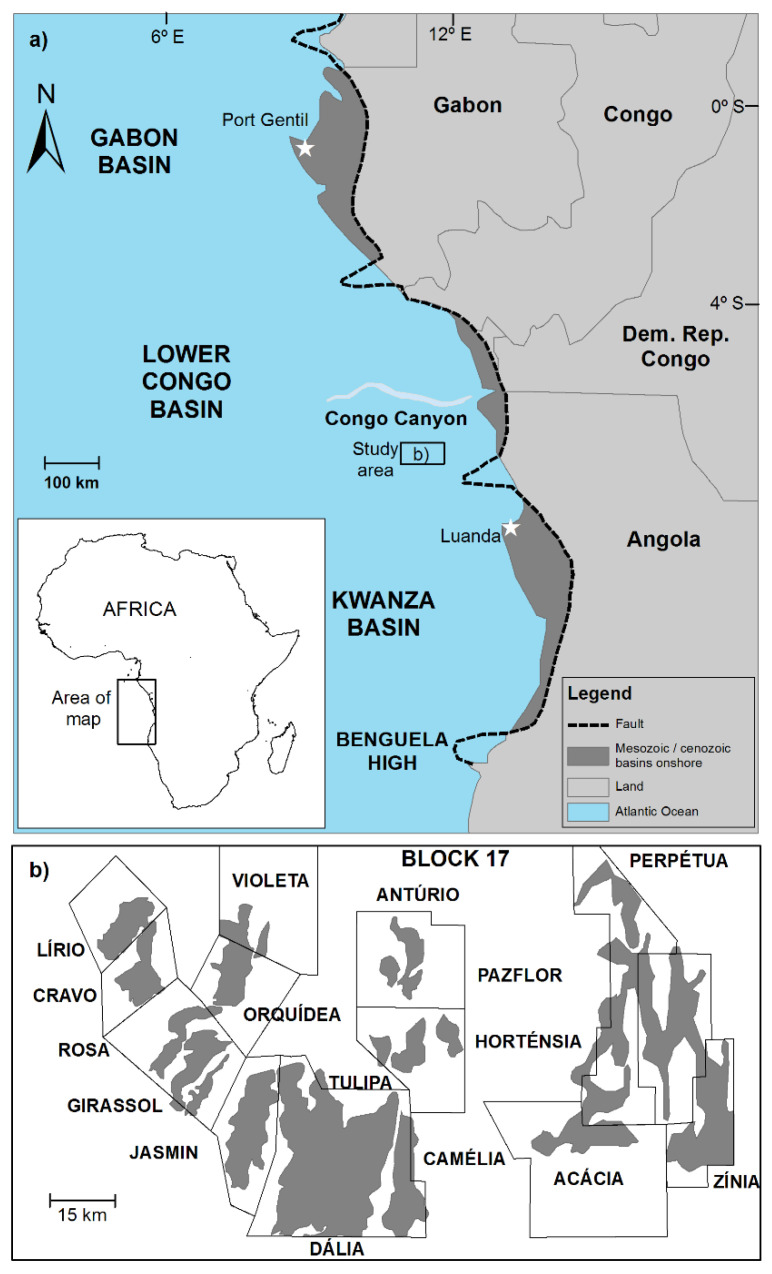
(**a**) Map showing the location of the study area in Africa; and (**b**) zoom revealing the location of the fields.

Angola is one of the largest crude oil producers in Sub-Saharan Africa. The country produces about 1.8 million barrels per day after a boom from 2002 to 2008 as its deepwater fields began to take off. Currently, crude oil production comes almost entirely from offshore fields off the coast of Cabinda and deepwater fields in the Lower Congo Basin (LCB). Angola began producing oil in 1956, when the company Petrofina discovered the first accumulation near Benfica in the Kwanza Basin, and has estimated crude oil reserves of 13 billion barrels. Most of the proved reserves are located in the offshore parts of the Lower Congo and Kwanza basins, which developed during the Late Jurassic and Neocomian times on the conjugated margins of Africa and Brazil. The study area is located within the sedimentary LCB (Figure 1a), that is about 250 km wide and located between the Mayumba apron in Gabón and the Ambriz Arch in Angola. Onshore exploration of the Lower Congo Basin began in 1966 and the first subcommercial discovery was at Cabeça de Cabra in 1968. Further, from 1969 to 1975, the company Texaco began offshore works in the Lower Congo Basin (LCB). Since 1978, the Angolan National Oil Company (Sociedade Nacional de Combustiveis de Angola) established the division of conventional offshore operations into 13 blocks. Additional oil reserves are also found increasingly distant from the coastline and, therefore, exploration licenses have taken the search for oil out to waters 1500 m depth and reserves buried up to 5500 m below the seabed on Angolan territory during the last three decades [1]. 

Block 17, Angola’s leading area with production at 600,000 barrels per day, covers an area of about 4000 km^2^ and is located around 230 km northwest of Luanda (Figure 1b); it has estimated oil reserves over 4 billion barrels. In Block 17, the petroleum industry had to find Paleogene oil deposits by 800 to 1600 m of water and then it had to drill a further 1500 to 2500 m from the seabed. GirRi (Girassol, Rosa, Jasmín, Antúrio, and Tulipa fields), Dália (the actual Dália and Camélia oilfields), Pazflor (Perpétua, Horténsia, Acácia, and Zínia fields), and the most recent CLOV (Cravo, Lírio, Orquídea, and Violeta oilfields) are the producing deepwater developments in Block 17 since 2001, 2006, 2011, and 2017. The GirRi, Dália, Pazflor, and CLOV projects lie about 120 to 150 km off the Angolan coast and most of them produce oil from Oligocene and Miocene Malembo reservoirs, with the exception of Dália that only produces oil from the Miocene upper Malembo reservoirs through at least 20 wells. GirRi (Girassol Resources Initiatives), Pazflor, and CLOV have estimated reserves around 2000, 700, and 500 million barrels, respectively, producing approximately 200,000, 205,000, and 160,000 barrels per day (92, 32, and 75% of which come from the Oligocene lower Malembo reservoirs) extracted from about 50, 34, and 19 producing wells [2].

Two main producing trends have been discovered in LCB. The first, discovered in Block 0 in Cabinda, produces from Cretaceous reservoirs in water depths less than 200 m; the second and most important, produces from Paleogene turbidite sand systems in water depths between 200 and 2000 m. Source rocks occur in three separate intervals: Early Cretaceous Bucomazi, Late Cretaceous Iabe, and Paleogene formations [3]. The lacustrine, organic-rich source rocks in the Bucomazi Formation generally contain type I kerogen and have a total organic carbon (TOC) average value exceeding 5 weight percent (wt.%), although the uppermost horizons of this latter unit contain type II kerogen averaging 2–3 wt.%TOC [4]. Late Cretaceous marine marls and shales from Iabe Formation contain mostly type II kerogen with TOC contents greater than 2 wt.% [5], yielding crude oils of single and/or mixed source provenance [6]. The Paleocene to Eocene marine shales of the Landana Formation have TOC values from 1 to 5 wt.%, while the Oligocene to Miocene Malembo Formation usually has TOC values between 1 and 5 wt.% TOC in the form of type II kerogen. However, these latter source rocks are generally immature [4]. Geochemical modeling suggests that Bucomazi and post-Albian source rocks (offshore LCB) began generating oil in the early late Cretaceous and middle Miocene, respectively, continuing to the present [7]. Last, Oligocene-Miocene Malembo turbidite reservoirs are exceptional [8].

In light of the above, this work was carried out using a set of six oil samples. These were taken from the lower and upper Malembo reservoirs in the offshore Block 17. The study aims to investigate the origin and thermal maturity of oil samples, as well as to assess the level of biodegradation.

## 2. Materials and Methods

### 2.1. Geological Settings

**Figure 2:**
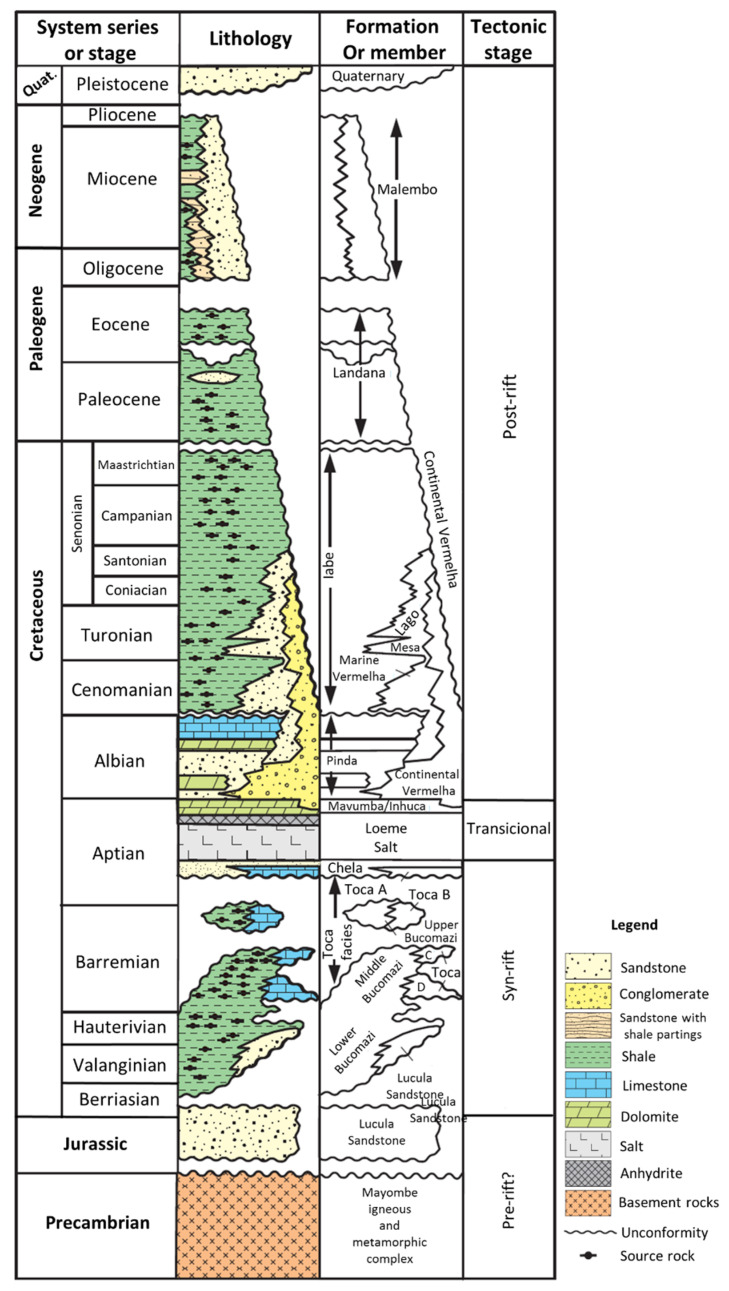
Stratigraphic column in the Lower Congo Basin.

The N–S-striking 200-km-long LCB extends from the coastline in the east to the escarpment at the base of the Angolan slope in the west [9]. LCB is one of the numerous salt sub-basins that developed during the opening of the West African passive margin in the early Cretaceous [10]. Tithonian to early Aptian rifting was followed by a massive late Aptian salt sequence named Loeme Formation ([11], see Figure 2). Among the pre-salt layers, the Bucomazi Formation is characterized by a lacustrine sedimentation deposited during Barremian to middle Aptian times, followed by the deposition of a thin layer of marine sediments known as the Chela layer [12]. Subsequent to evapotitic sedimentation, a carbonate/siliciclastic sequence was deposited so that the post-salt cover evolved from carbonate deposition represented by the Albian shallow-marine Pinda Group to the marine siliciclastic Iabe Formation in the Cenomanian-Maastrichtian ([13], Figure 2). The Paleocene-Eocene Landana Formation was also deposited in deep marine settings with low sedimentation rates, until the beginning of the ancestral Congo turbiditic sedimentation system in LCB since the Oligocene [14], resulting in the Miocene siliciclastic Malembo Formation and the Congo deep-sea fan [15]. From Pliocene to the present, the shelf and the slope are deeply incised by a canyon that directly connects the Congo River with the basin floor. The post-Miocene interval is considered as a good seal above the Malembo Formation [16]. 

The Congo Basin is one of several sub-basins in the Aptian Salt Basin Mega-System developed along the West Coast of Africa, which is edged to the North by the Gabon Basin and to the South by the Kwanza Basin. The so-called Lower Congo Basin, that belongs to the Congo Basin, corresponds to the portion located in the North part of Angola. The Congo Basin represents a rift basin formed during the South Atlantic Opening, as Africa and South America began to detach. The rifting process started in the late Jurassic and early Cretaceous (150–140 Ma) and ceased around the late Barremian and early Aptian (127–117 Ma) [14,17,18,19,20,21,22]. In fact, the rift basins were separated from the early Cretaceous South Atlantic Ocean by the Walvis Ridge located off the Namibia coast. However, during the Aptian time, the Walvis Ridge was subsided below sea level allowing the South Atlantic Ocean to invade the rift basins to the North, which promoted a change in depositional environmental conditions from continental (fluvio-lacustrine) to marine. This transition of environmental conditions is clearly recorded by late Aptian evaporites sequence. Additionally, it seems that the West African coast was subjected to several rift episodes showing different durations, which induced the seafloor spreading between Africa and America. As is well known, the sedimentation processes can be strongly controlled by tectonic events, and considering that the spatial and temporal distribution of these rift episodes can vary, and did so across and along the African coast. Therefore, the interaction between subsidences and uplifts, developed at different stages, allowed the development of a complex stratigraphic sequence, in response to changes in sediments inputs, physiography, relative sea-level and consequently environmental conditions. Although, the complexity of tectonic and sedimentary processes established during rifting phase, post-rift phase embodies a complex evolution strongly influenced by salt movements and raft structures [4,23]. Tectonic and sedimentary basin evolution of Congo Basin, as well as all basins in Angolan margin, developed during Mesozoic and Cenozoic followed four classical stages of passive margins phases, namely pre-rift, syn-rift, transition and post-rift (Figure 2) [4,5,14].

The pre-rift phase (late Proterozoic to late Jurassic) is characterized by an extreme peneplanation process and deposits are represented by a sandy fluvial-lacustrine sequence [5,14,24], which directly overlies faulted metamorphic and granitic basement prior to major continental rifting. The syn-rift phase (late Jurassic to early Aptian) is, usually, recognized as being three stages, namely syn-rift I, syn-rift II and sag [15,25]. Each rift stage resulted in the generation of deep and underfilled lacustrine small basins; nevertheless, the first phase (syn-rift I) is established as the most important one. The syn-rift I (Berriasian to Valanginian) was strongly affected by the development of horst and graben basins and is characterized by a continental fill commonly associated to fluvial and alluvial fan systems [19,21,22]. The syn-rift II (Hauterivian to Early Barremian) represents the stage in which the basin was submitted to an extension large enough to allow the development of a big lacustrine system. This sequence (bottom to top) is characterized by sandstones, as a result from the structural reactivation of the basin, organic-rich shales and the top unit called Toca (Figure 2), considering the overfilling of the basin, is represented by claystones interbedded with limestones and calcareous sandstones. After this lacustrine system, another structural reactivation took place in the basin promoting the erosion of the upper layers of the lacustrine system previously deposited. This unconformity has been reported by several authors [4,13,15,23] and represents the beginning of the sag event (late Barremian to early Aptian, Figure 2) which is characterized by an early Aptian sea-level fall and the sandy bodies identified are associated with flood pulses (Chela Formation). 

The transitional phase (middle Aptian to early Albian) represents the transition from a continental-lacustrine to a restricted marine environment, which was subjected by evaporation processes leading to the formation of a salt sequence mainly composed of halite [4,13,21,23]. Finally, the post-rift phase (early Albian to present) started with a breakup unconformity just above the salt and is characterized by thick marine successions. The post-rift (also generally referred to as the post-salt) has experienced a complex history of structural deformation. In fact, this phase has been strongly influenced by raft tectonics, which allowed the development of small basins (raft depocentres) due to the ductile behavior of the salt deposited during the Aptian time. However, these rafting processes, which were present during the whole post-rift phase, showed stronger reactivations at the beginning of the sequence [23,25,26]. The post-rift phase is characterized by two major stratigraphic units, which reflects a major change in ocean circulation and climate. The two mega units are separated by a depositional hiatus produced by the global lowering of sea level in the early Oligocene. The first stratigraphic unit (from upper Cretaceous to early Oligocene time) is characterized by an aggradational carbonate/siliciclastic ramp, which developed due to low-amplitude/low-frequency sea-level changes and a uniform climate (greenhouse period). The second stratigraphic unit (early Oligocene to recent) is characterized by a progradational terrigenous sequence, which reflects high-amplitude/high-frequency sea-level changes and alternating dry and wet climate conditions [25,27,28].

### 2.2. Sampling, Chemical Analysis and QEDA 

Three oil samples (collected from Tulipa, Acácia, and Orquídea wells) from the lower Malembo reservoirs and the others three oil samples (collected from Horténsia, Rosa, and Dália wells) from the upper Malembo reservoirs in the Block 17 area were analyzed (Table 1 and Figure 1b). API gravities of sampled oils were determined using the D287-92 standard [29]. An aliquot of each sample was fractionated into saturates (SAT), aromatics (ARO), and resins plus asphaltenes (POL). Firstly, asphaltenes were separated with *n*-heptane in a 1:40 *v*/*v* ratio following the procedure established by Speight [30]. Later, maltenes were separated into saturated, aromatic, and resins by liquid chromatography in columns filled with silica gel and alumina [31]. Solvents used to elute these fractions were *n*-hexane, dichloromethane/hexane (7:3 *v*/*v*), and dichloromethane/methanol (1:1, *v*/*v*), respectively. 

Saturated and aromatic compounds were analyzed by gas chromatography-mass spectrometry (GC/MS). The GC-MS analysis was carried out on a 7890A GC System (Agilent Technologies) coupled to a 5975C Inert XL MSD with Triple-Axis Detector (Agilent Technologies). Helium was used as carrier gas. Gas chromatography was performed on a capillary column DB-5ms (60 m × 0.25 mm i.d. × 0.10 μm film thickness), from Agilent Technologies. The initial oven temperature was 50 °C (held for 2 min) and ramped at 2.5 °C to reach 300 °C (held for 70 min). The mass spectrometer was operated in electron impact mode (EI) at 70 eV at daily calibration autotuning conditions. The chromatograms were acquired in full-scan and single-ion monitoring modes (mass range acquisition was performed from *m/z* 45 to 500).

Quantitative extended diamondoid analysis (QEDA) was performed on maltenes (saturated fraction) from two representative oil samples using gas chromatography-triple quadrupole mass spectrometry [32,33]. Furthermore, once hydrous pyrolysis of asphaltenes of these oil samples was conducted following the procedures described in [34], QEDA fingerprints were also observed in the respective asphaltene hydrous pyrolysates. Four deuterated internal standards were used to quantify triamantane molecule and extended polimantanes, namely, triamantane-d4 and cyclohexamantane-d8, as well as tetramantane-d6 and pentamantane-d6 for the four-cage (T1, T2, and T3) and five-cage (P1, P2, P3, and P4) non-enantiomorphic isomers. Shapes of tetra-, penta-, and hexamantane (H1) compounds measured are shown in [35].

## 3. Results and Discussion

### 3.1. Bulk Geochemical Data 

Bulk data including the SARA fractionating and API gravity of each sampled oil are shown in Table 1. The oil samples from the upper Malembo reservoir showed API gravities in the 20–21° range and similar compositions: SAT ranging between 59 and 61%, ARO ranging from 25 to 27%, and POL about 14%. The samples from the lower Malembo reservoir showed API gravities, SAT, ARO, and POL weight percent in the 35–37°, 69–70%, 21–22%, and 9–10% ranges, respectively. All these values are typical of apparently normal oils according to [36]. Differences in these datasets could be explained by biodegradation [37].

### 3.2. In-Reservoir Biodegradation 

**Figure 3:**
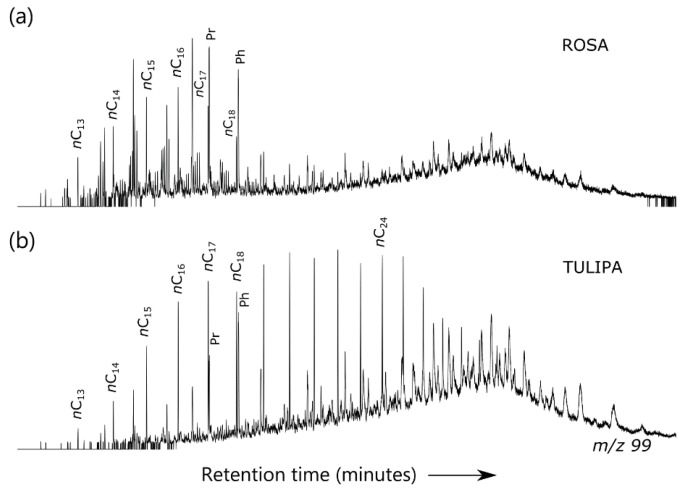
(**a**) and (**b**), respectively, *m/z* 99 ion chromatograms showing saturated hydrocarbon signals for typical oils from the lower and upper Malembo reservoirs.

**Figure 4:**
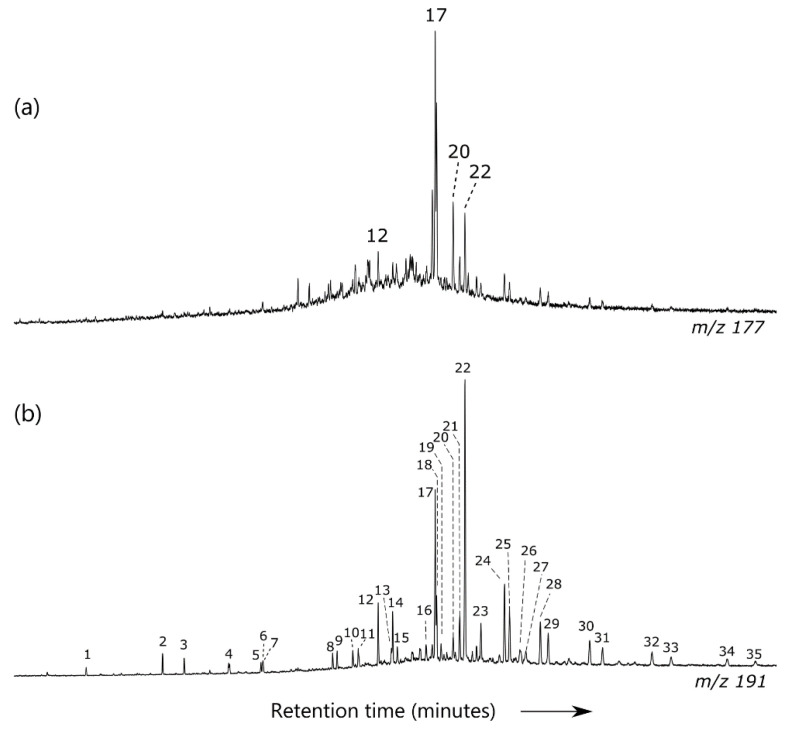
(**a**) and (**b**), respectively, *m/z* 177 and *m/z* 191 ion fragmentograms for the saturated fraction showing the triterpane distribution for a representative oil sample.

The *m/z* 99 ion chromatograms of the saturate fraction of two representative oil samples are shown in Figure 3, denoting the presence of an “unresolved complex mixture” associated with naphthenic groups generated through biodegradation processes [38]. API gravities below 35° can also be a sign of biodegradation [39], and/or can be indicative of a mixture of at least two oil charges. Twenty-five norhopanes are absent in all the studied samples (*m/z* 177 fragmentograms of representative sampled oils are shown in Figure 4a; peak identifications are in the Supplementary Material section). The absence of these biomarkers precludes severe biodegradation [40].

A more detailed biodegradation scheme can be established by using PM [41] and Manco [42] scales. There is evidence of partial depletion of *n*-alkanes and no or negligible alteration of isoprenoid alkanes in lower Malembo oils analyzed, indicating a PM level of 1. The other samples have a notable absence of *n*-alkanes, which is coherent with PM degradation level of 2. Differences in the degradation degrees of normal and isoprenoid alkanes in the sampled oils could be explained due to multiple reasons, such as the combination of distinct reservoir temperatures and microbial communities, the relative rates of biodegradation or the influx of fresh oil within reservoir compartments [43,44].

### 3.3. Precursor Organic Matter and Depositional Environments

A number of molecular indicators have been calculated to infer characteristics of the paleoenvironment and the type of organic matter. The *n*-alkane patterns for the lower Malembo samples were very similar among them, bimodal with maximum peaks in *n*-C_17_ and *n*-C_22_ (Figure 3), and apparently characteristic of lacustrine precursor organic material [45]. Pristane-to-phytane (Pr/Ph) values lie in the 1.2–1.7 range (Table 2), which is coherent with organic matter deposited under suboxic to dysoxic conditions [36]. The Pr/Ph ratio may be influenced by thermal maturity and other processes [46], but it can be used here to denote the depositional setting. Relatively high Pr/*n*-C_17_ (≥0.5) and Ph/*n*-C_18_ values can also denote slight biodegradation processes [45]. DBT/P ratios are clearly lower than 1 in all the studied samples. The combination of the DBT/P and Pr/Ph ratios suggests a lacustrine sedimentary environment [47]. 

**Figure 5:**
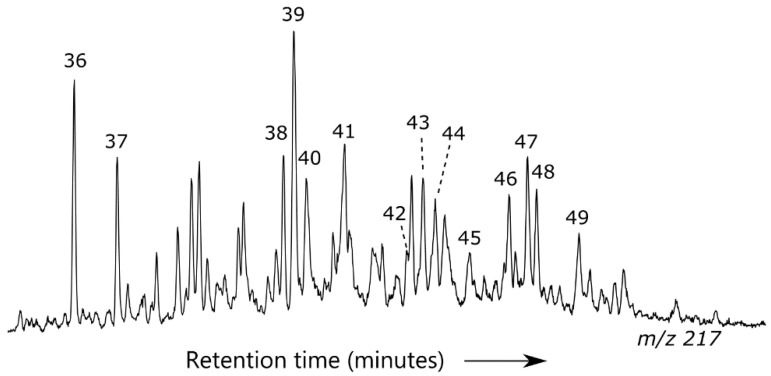
Example of *m*/*z* 217 ion chromatogram showing sterane distribution for a representative oil sample.

Oil samples showed similar triterpane and sterane distributions (Figure 4b and Figure 5). Any *m/z* 191 ion chromatogram of the saturated fraction exhibits a high relative abundance of the C_23_ with respect to the C_24_ and other homologues (Table 2), thereby indicating an organic material precursor deposited in a lacustrine environment [45]. Table 2 shows C_29_/C_30_ hopane ratios lower than 0.8, C_26_/C_25_ cheilanthane values superior to 1, C_31_R/C_30_ hopane ratios inferior to 0.3, low sterane-to-hopane values, and ratios of 18α(H)-22,29,30 trisnorneohopane to 17α(H)-22,29,30 trisnorhopane (Ts/Tm) exceeding 1, which may indicates that these oils were originated from lacustrine source rocks deposited under low-oxygen conditions [48,49]. Furthermore, a representative *m/z* 217 ion chromatogram of the saturated fraction exhibits similar abundances of C_27_ regular steranes compared to the C_28_ and C_29_ counterparts; this does not preclude oils formed from lacustrine sources [50]. A siliciclastic source facies for the studied oils is also supported by high values (>0.5; Table 2) of the ratio of diasteranes to regular steranes [51]. However, oleanane-type compounds are present in the oil samples (Figure 4b), these biomarkers are related to angiosperms and can be used as Paleogene age indicators [52]. Thus, a second oil charge was recorded for all sampled oils and is inferred to originate from Paleogene terrigenous Malembo Formation source rocks.

As for the methyldibenzothiophene (MDBT) isomers, all the analyzed samples showed the typical siliciclastic lithology distribution patterns (i.e., the 1-methyl isomer being the lowest; [53]), which can also be indicative of argillaceous source facies [49].

### 3.4. Thermal Maturity 

**Figure 6:**
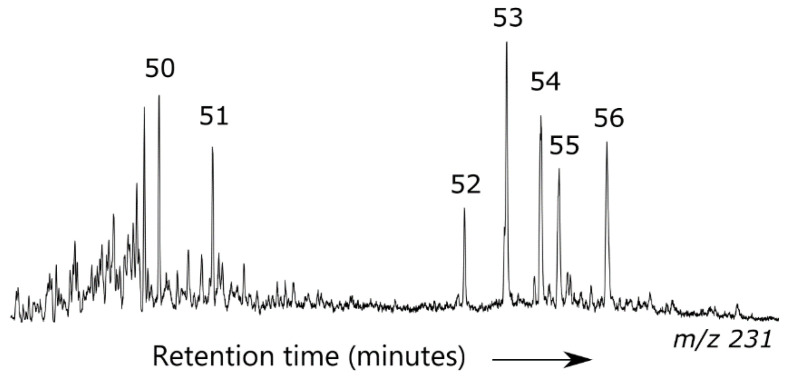
Representative *m*/*z* 231 ion chromatogram showing triaromatic steroids for the aromatic fraction of oil samples from the Block 17.

Molecular maturity parameters for saturated and aromatic fractions of the oil samples are shown in Table 3. Sterane isomerization parameter (%ββ) is described in the literature to rise from 0–0.5 to 0.7 with increasing thermal maturity [54]. The samples showed %ββ values of 51–59% (Table 3), which would be consistent with a maturation level equivalent to the onset of the peak of the oil window [55]. The triaromatic steroid peaks (Figure 6) were examined and similar values of the triaromatic steroid ratio for the oil samples (0.39; Table 3) were obtained in all the samples, which would indicate maturity levels before the peak oil generation [56]. Table 3 shows the methylphenanthrene index values (MPI-1; [57,58]) corresponding to all the oil samples. The studied oils showed MPI-1 values around 0.9 and MPI-1-based calculated vitrinite reflectance data (%Rc_3_) about 0.95%. These values suggest maturation levels beyond the peak of the oil window and they are high when compared to the other calculated vitrinite reflectance data. This latter observation would corroborate that the MPI-1 ratio is not a useful indicator of maturation level for oils derived from predominantly lacustrine organic matter at early maturities [59].

### 3.5. Geochemical Correlations

An hierarchical cluster was performed based on 10 source-related biomarker parameters (Pr/Ph, 26/25T, 24/23T, Ts/Tm, 29/30H, DBT/P, %27ST, ST/H, %29ST, diasterane ratio, and 31R/30H), following the Ward algorithmic method [60]. The Euclidean distance was used to measure divergence [61]. A single genetic oil family is confirmed by the agglomerative hierarchical clustering using the proximity procedure. 

Integration of all results seems to indicate that the oil samples consist of a mixture composed of a pre-existing biodegraded oil charge, which sourced from a lacustrine facies of the Bucomazi Formation source rocks, whilst a fresh pulse originated from a second terrestrially-derived source facies of the Malembo Formation. 

**Figure 7:**
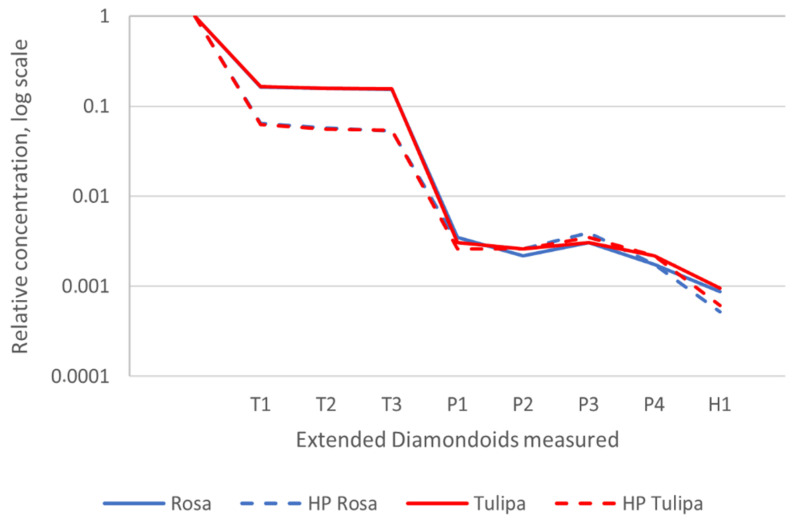
Fingerprints of higher diamondoids by QEDA for Rosa (red in the graphics) and Tulipa (in blue color) oils (denoted by continuous lines) and respective hydropyrolysates generated from asphaltenes (dash lines). Note: The abundances of other polymantanes are relative to that of triamantane, which is set at one [35].

Extended diamondoid fingerprints can be used for oil-oil correlations because higher diamondoids are present in almost all oils, regardless of microbial alteration or thermal maturation (e.g., [39,62]). QEDA fingerprints from triamantane and eight higher diamondoids [35] in two representative sampled oils were observed (Figure 7). Rosa and Tulipa oils were not differentiated by QEDA. However, according to the work by Moldowan [63], the QEDA method can be used not only for a direct correlation between oils, but also between oils and their asphaltenes. This method is particularly powerful to determine oil mixtures and identify the co-sources of the mixtures. Here, we compared QEDA fingerprints between Rosa and Tulipa (maltenes) and the respective asphaltene hydrous pyrolysis samples, which revealed differences between fingerprints for saturated fractions of the selected oils and their respective asphaltene hydrous pyrolysates (Figure 7), providing clear evidence of mixing with very similar proportions of the lacustrine-sourced Bucomazi Formation and more terrigenous-sourced Malembo Formation oil types in both representative samples. 

## 4. Conclusions

Analyzed oil samples from six fields in the Angolan Block 17 originated from the same source rocks (the lower Cretaceous Bucomazi and Paleogene Malembo formations) consist of a mixture of two generation pulses of hydrocarbons. Biomarker data indicate that the first pulse originated from the Bucomazi Formation lacustrine facies, whilst the second one was generated from the more terrestrially derived facies of the Malembo unit. All the study oils belong to the same genetic type. Differences in the PM levels of biodegradation within the study oils could be explained by factors such as reservoir temperature and/or microbial communities.

## Figures and Tables

**Table 1:** API gravities and SARA fractionating for sampled oils.

Well	Reservoir	API	SAT	ARO	POL
Horténsia	Upper Malembo	20.2	60	26	14
Dália	Upper Malembo	21.4	61	25	14
Rosa	Upper Malembo	21.0	59	27	14
Tulipa	Lower Malembo	35.1	69	21	10
Acácia	Lower Malembo	35.8	70	21	9
Orquídea	Lower Malembo	36.3	69	22	9

SAT = saturates, ARO = aromatics, and POL = polar compounds.

**Table 2:** Geochemical indicators of source type and *depositional* environment for saturated and aromatic fractions in oils analyzed.

Sample	%27ST	%28ST	%29ST	Ts/Tm	Ph/*n*C_18_	Pr/Ph	29/30H	31R/30H	26/25T	24/23T	ST/30H	DBT/P	Dia/ST
Horténsia	35	32	33	1.38	4.63	1.42	0.57	0.24	1.14	0.76	0.22	0.15	0.53
Dália	36	31	33	1.33	4.20	1.51	0.53	0.23	1.12	0.75	0.24	0.13	0.51
Rosa	35	31	34	1.21	4.35	1.68	0.59	0.20	1.12	0.76	0.20	0.11	0.51
Tulipa	35	31	34	1.41	1.30	1.20	0.51	0.21	1.14	0.72	0.23	0.13	0.53
Acácia	35	32	33	1.34	1.24	1.30	0.58	0.22	1.13	0.73	0.23	0.12	0.52
Orquídea	36	31	33	1.27	1.19	1.43	0.55	0.22	1.15	0.74	0.21	0.14	0.52

24/23T = C_24_-cheilanthane/C_23_-cheilanthane; 29/30H = 30-norhopane/hopane; Pr/*n*C_17_ = pristane/*n*-heptadecane; DBT/P = dibenzothiophene/phenanthene; 31R/30H = homohopane 22R/hopane; %27ST = percentage of C_27_ regular steranes; 26/25T = C_26_-tricyclopolyprenanes/C_25_-tricyclopolyprenane; ST/30H = ratio of C_29_-regular steranes to C_30_-hopane; Ts/Tm = 18α(H)-22,29,30 trisnorneohopane/17α(H)-22,29,30 trisnorhopane; Pr/Ph = pristane/phytane; and Dia/ST = diasterane ratio or C_27_-diasteranes/C_27_-regular steranes.

**Table 3:** Some maturity-related molecular parameters for saturated and aromatic fractions in the oil samples.

Sample	%ββ	Rc_1_	TA	Rc_2_	MPI-1	Rc_3_
Horténsia	56	0.63	0.33	0.63	0.92	0.95
Dália	57	0.64	0.32	0.62	0.90	0.94
Rosa	55	0.60	0.33	0.63	0.92	0.95
Tulipa	56	0.61	0.34	0.65	0.91	0.95
Acácia	56	0.61	0.32	0.62	0.91	0.95
Orquídea	57	0.63	0.35	0.66	0.93	0.96

Notes: %ββ = ratio (%) of C_29_ isosteranes (20*S* + 20*R*) to C_29_ regular steranes (20*S* + 20*R*); %Rc_1_ = 0.01·(33.33 + 0.487·%20S); TA = C_20_ homologue to C_20_ plus C_28_ 20R triaromatic steroid ratio; Rc_2_ = 0.37 + 0.7·TA; MPI-1 = 1.5·(2-MP + 3-MP)/(P + 1-MP + 9-MP); and Rc_3_ = 0.4 + 0.6 MPI-1.

## Supplementary Materials

The following are available online at https://www.mdpi.com/1660-4601/17/19/7204/s1, Table S1: Main saturated and aromatic biomarkers identified in the fragmentograms.
