# Supplementary material for: Classical Biomarker and Quantitative Extended Diamondoid Analysis Fingerprints for Crude Oils from Deepwater Developments in Block 17, Lower Congo Basin, Angola
Source: Int J Environ Res Public Health. 2020 Oct 1;17(19):7204. doi: 10.3390/ijerph17197204 (PMC7579521; doi:10.3390/ijerph17197204)
Supplement: Supplementary file 1 [file ijerph-17-07204-s001.pdf]

Supplementary material for the manuscript: **Classical biomarker and QEDA fingerprints for crude oils from deepwater developments in Block 17, Lower Congo Basin, Angola**

Annex: Main saturated and aromatic biomarkers identified in the fragmentograms.

| | | | |
|---|---|---|---|
| 1 | $C_{21}$-Tricyclic terpane | 29 | 17α(H),21β(H)-29-Bishomohopane 22R |
| 2 | $C_{23}$-Tricyclic terpane | 30 | 17α(H),21β(H)-29-Trishomohopane 22S |
| 3 | $C_{24}$-Tricyclic terpane | 31 | 17α(H),21β(H)-29-Trishomohopane 22R |
| 4 | $C_{25}$-Tricyclic terpane 17R+17S | 32 | 17α(H),21β(H)-29-Tetrahomohopane 22S |
| 5 | $C_{26}$-Tricyclic terpane 17R | 33 | 17α(H),21β(H)-29-Tetrahomohopane 22R |
| 6 | $C_{24}$-Tetracyclic terpane | 34 | 17α(H),21β(H)-29-Pentahomohopane 22S |
| 7 | $C_{26}$-Tricyclic terpane 17S | 35 | 17α(H),21β(H)-29-Pentahomohopane 22R |
| 8 | $C_{28}$-Tricyclic terpane 17R | 36 | 13β(H),17α(H)-Diacholestane 20S |
| 9 | $C_{28}$-Tricyclic terpane 17S | 37 | 13β(H),17α(H)-Diacholestane 20R |
| 10 | $C_{29}$-Tricyclic terpane 17R | 38 | 5α(H),14α(H),17α(H)-Cholestane 20S* |
| 11 | $C_{29}$-Tricyclic terpane 17S | 39 | 5α(H),14β(H),17β(H)-Cholestane 20R* |
| 12 | 18α(H)-22,29,30-Trisnorneohopane | 40 | 5α(H),14 β(H),17β(H)-Cholestane 20S |
| 13 | $C_{30}$-Tricyclic terpane 17R | 41 | 5α(H),14α(H),17α(H)-Cholestane 20R |
| 14 | 17α(H)-22,29,30-Trisnorhopane | 42 | 5α(H),14α(H),17α(H)-Ergostane 20S |
| 15 | $C_{30}$-Tricyclic terpane 17S | 43 | 5α(H),14β(H),17β(H)-Ergostane 20R* |
| 16 | 18α(H)-24,28-Bisnoroleanane | 44 | 5α(H),14β(H),17β(H)-Ergostane 20S |
| 17 | 17α(H),21β(H)-30-Norhopane | 45 | 5α(H),14α(H),17α(H)-Ergostane 20R |
| 18 | 18α(H)-30-Norneohopane | 46 | 5α(H),14α(H),17α(H)-Stigmastane 20S |
| 19 | 18α(H)-28-Noroleanane | 47 | 5α(H),14β(H),17β(H)-Stigmastane 20R |
| 20 | 17β(H),21α(H)-30-Normoretane | 48 | 5α(H),14β(H),17β(H)-Stigmastane 20S |
| 21 | 18α(H)-Oleanane | 49 | 5α(H),14α(H),17α(H)-Stigmastane 20R |
| 22 | 17α(H),21β(H)-Hopane | 50 | 20-triaromatic steroid hydrocarbon |
| 23 | 17β(H),21α(H)-Moretane | 51 | 21-triaromatic steroid hydrocarbon |
| 24 | 17α(H),21β(H)-29-Homohopane 22S | 52 | 26-triaromatic steroid hydrocarbon 20S |
| 25 | 17α(H),21β(H)-29-Homohopane 22R | 53 | 26 (20R) + 27 (20S)-triaromatic steroid* |
| 26 | Gammacerane | 54 | 28-triaromatic steroid hydrocarbon 20S |
| 27 | 17β(H),21α(H)-29-Homomoretane 22S+22R | 55 | 27-triaromatic steroid hydrocarbon 20R |
| 28 | 17α(H),21β(H)-29-Bishomohopane 22S | 56 | 28-triaromatic steroid hydrocarbon 20R |

*Peak co-elution
